# Are we missing the diagnosis of depression in patients with rheumatoid arthritis at a tertiary care facility?

**DOI:** 10.12669/pjms.332.11856

**Published:** 2017

**Authors:** Ammara Masood, Babur Salim, Amjad Nasim, Ziaullah Khalid, Amir Afzal

**Affiliations:** 1 Dr. Ammara Masood, MRCP, SCE (Rheumatology). Dept. of Rheumatology, Fauji Foundation Hospital, Rawalpindi, Pakistan; 2 Dr. Babur Salim, FCPS(Med), FCPS (Rheumatology). Dept. of Rheumatology, Fauji Foundation Hospital, Rawalpindi, Pakistan; 3 Dr. Amjad Nasim, FCPS (Med). Dept. of Rheumatology, Fauji Foundation Hospital, Rawalpindi, Pakistan; 4 Dr. Ziaullah Khalid, MBBS. Dept. of Rheumatology, Fauji Foundation Hospital, Rawalpindi, Pakistan; 5 Amir Afzal, Statistician. Dept. of Rheumatology, Fauji Foundation Hospital, Rawalpindi, Pakistan

**Keywords:** BDI, Depression, mHAQ, Rheumatoid Arthritis

## Abstract

**Objectives:**

To determine if we are missing clinical depression in patients with Rheumatoid Arthritis and its relationship with functional disability and level of formal education in such patients.

**Methods:**

The data for this cross-sectional, analytical study was gathered from May 2015 till December 2015 and comprised of 128 with Rheumatoid arthritis diagnosed according to ACR/EULAR 2010 criteria. The study was conducted at Fauji Foundation Hospital Rawalpindi. Functional status was assessed with Modified Health Assessment Questionnaire (mHAQ) and Beck’s Depression Inventory (BDI) was used for evaluation of symptoms of depression. The relation between depression, functional disability and educational status was established using Pearson correlation coefficient.

**Results:**

The study included 128 patients with no previous diagnosis of depression. 122 (95.3%) were females and 6 (4.7%) were males. The mean age was 51.75 ± 9.25 years. Mean duration of disease was 8.95 ± 7.1 years. According to this study, the diagnosis of clinical depression was missed in 47.7% of patients with Rheumatoid Arthritis who had been under regular follow up at a tertiary care facility. About 18% were keen to seek professional help for depressive symptoms while 62.6% had functional disability (mild – severe). There is a positive correlation with BDI (Pearson’s correlation +1) and functional disability. No correlation could be established between level of education and depression as out of 79 (61.7%) patients with no basic education, 45.5% had depression. In remaining 49 (38.2%) patients, with some formal education, 51.3% had clinical depression.

**Conclusion:**

Almost half of the patients with Rheumatoid Arthritis coming to a tertiary care set up had clinical depression but were never diagnosed or referred to a Psychiatrist. There is a positive correlation between depression and functional disability; however no statistically significant correlation could be established with the level of formal education. The study further emphasizes the importance of early recognition and swift referral of such patients to a psychiatrist since it is known to improve both treatment outcomes and functional status.

## INTRODUCTION

Rheumatoid Arthritis (RA) is an autoimmune, chronic inflammatory disease characterized by joint swelling, joint tenderness, and destruction of synovial joints, leading to severe disability and premature mortality.^1^ It continues to cause modest global disability, with severe consequences in the individuals affected. Rheumatoid arthritis has a global prevalence of 0.24%, with no discernible change from 1990 to 2010.^2^ In urban population of Southern Pakistan, Karachi, prevalence is 0.14% where as in Northern Pakistan 0.55% population suffers from Rheumatoid arthritis.^3^ In India, prevalence is reported at 0.75% which is comparable to figures from Pakistan.^4^

Almost 450 million people in the world suffer from a mental or behavioral disorder.^5^ Lifetime prevalence varies widely, from 3% in Japan to 17% in the US. In most countries the number of people who would suffer from depression during their lives falls within an 8–12% range.^6^,^7^

There is paucity of data with regards to epidemiology of depression in Pakistan, however a study involving three capital cities reported that amongst local household, 45.98% are suffering from depression or having symptoms of depression.

Depression and anxiety are highly prevalent in RA. A recent meta-analysis reporting a 16.8% point prevalence of depression, diagnosed via clinical interview and an estimated prevalence of around 14.8 to 33.8%.^8^ RA can cause a negative impact on the psychological health of patients leading to mental distress and depression. Such patients commonly experience a life of dissatisfaction and psychological distress and are five times more likely to suffer from depression compared to normal population.^8^

The mental health and psychological functioning of RA patients has most frequently been operationalized through measures of depression, anxiety and quality of life.^9^ Symptoms of depression and anxiety have implications for disease activity and despite well controlled inflammatory disease markers may indicate significant psychological morbidity and non-inflammatory pain, rather than true disease activity.^10^ There is substantial evidence to suggest that depression and anxiety can be effectively treated in physical conditions and may eventually improve disease outcomes.^11^

The association between disease activity outcomes and depression/anxiety is strong.^12^ That means if depression is ameliorated, it can improve over all patient global assessment scores in RA patients. Moreover in RA patients, with moderate to high disease activity, clinical remission reduces symptoms of depression/anxiety, and independently improves patient reported outcomes (PROs), thereby suppressing the negative impact of depression/anxiety on these measures.^13^ Thus depression and disease activity in RA are directly or indirectly have impact on global assessment scores.

It underscores the need of early identification and subsequent management of depression as part of patient’s comprehensive treatment plan. This study primarily aimed to identify patients with RA, visiting a tertiary care facility, in which clinical depression had been possibly missed. In that case we should focus on diagnosing depression earlier to achieve a better outcome.

## METHODS

The study was conducted at the Rheumatology Department of Fauji Foundation Hospital Rawalpindi, Pakistan. One hundred twenty eight patients with RA diagnosed on the basis of ACR/EULAR 2010 classification criteria^14^ were enrolled. It is a cross-sectional, analytical study. Data was collected from May 2015 till December 2015. All patients were older than 18 years and had RA for minimum of six months. They had more than four clinic visits and had no prior history of any psychiatric ailment or any other chronic illness besides RA. We excluded those patients who were coming for their first visit as we wanted to see in how many patients we were missing the diagnosis of depression. An informed consent was taken from all the patients and the study was approved by the local ethical committee.

Functional status was assessed with Modified Health Assessment Questionnaire (mHAQ),^15^ and Beck’s Depression inventory II (BDI) was used for evaluation of symptoms of depression.^16^ The questionnaires were filled by the patients. The relation between depression, functional disability and educational status was established using Pearson correlation coefficient. Severity of functional limitation in RA was assessed using modified health assessment questionnaire disability Index (mHAQ) with the following range of scores: Normal < 0.4, Mild 0.4-1.2, Moderate 1.3-1.8, severe> 1.8. Severity of depression was measured using Beck Depression Inventory II scale questionnaire with following range of scores: 0-13(no depression), 14-19(mild to moderate depression), 20-28 (moderate to severe depression) and 29- 63(severe depression).^16^

The relationship between depression and functional disability in Rheumatoid Arthritis patients was evaluated. Data was analyzed using SPSS 20 software. Variables included duration of disease, duration of treatment, disability, educational status, number of joints damaged and inflammatory markers (ESR). Data stratification was done according to these variables and frequencies were calculated. Correlation coefficient among dependent (depression) and independent (mHAQ) variables was calculated. Chi square test was applied to determine significant association between the frequencies of depression in patients with mild, moderate, and severe functional limitation. ‘p’ value of 0.05 or less was taken as significant.

## RESULTS

The study included 128 patients with no previous diagnosis of depression. 122 (95.3%) were females and 6 (4.7%) were males. The mean age was 51.75 ± 9.25 years. Mean duration of disease was 8.95 ± 7.1 years (Table-I).

**Table-I:** Demography.

*Variables*	*N*	*Mean*	*Std. Deviation*
Age (years)	128	51.75	9.25
Duration (years)	128	8.957	7.1892
No. of Joint Damage	128	3.063	3.8490
Duration of Treatment	128	6.723	5.7708
ESR	128	32.39	10.450
N	128		

**Fig.1:**
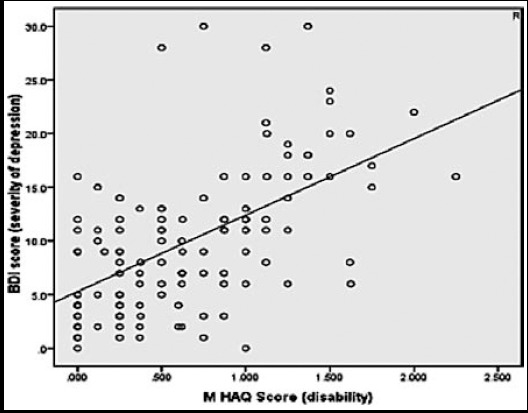
Correlation between severity of depression and mHAQ score.

**Fig.2:**
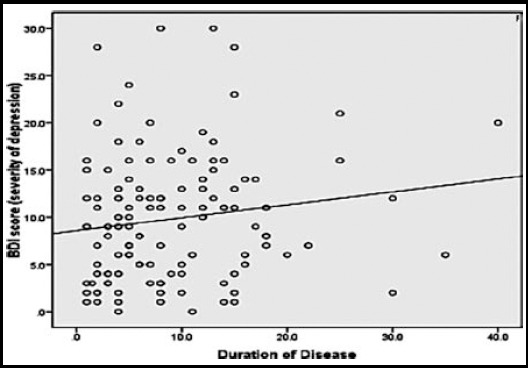
Comparison between severity of depression and duration of disease.

According to this study, 47.7% of patients were found to have depression of varying severity.62.6% had functional disability (mild to severe) and a positive correlation with BDI (Pearson’s correlation+1) as depicted in Fig.1. Out of 79 (61.7%) patients with no basic education 45.5% had depression. In remaining 49 (38.2%) patients, with some formal education, 51.3% had clinical depression. A linear relation was found between severity of depression and duration of disease (Fig.2). No correlation could be established between severity of depression and variables like education, family unit (joint or nuclear), ESR and duration of treatment. Only 18% of patients with clinical depression, when offered, were keen to seek professional help for depressive symptoms while 82% refused an opinion from a psychiatrist.

mHAQ was found to be positively correlated with severity of depression with r =0.32 and p value <0.001. A linear correlation was found when BDI II scores were compared with duration of disease (p=0.09) and number of damaged joints (p=0.02).

**Table:** Correlations

		*M HAQ*	*BDI score*
M HAQ Score	Pearson Correlation	1	0.571**
	Sig. (2-tailed)		0.000
	N	128	128
BDI score	Pearson Correlation	0.571**	1
	Sig. (2-tailed)	0.000	
	N	128	128

** Correlation is significant at the 0.05 level.

## DISCUSSION

RA is a chronic inflammatory joint disease associated with considerable comorbidity, which interferes with normal functioning, well-being and contributes to a decline in quality of life.^17^ Over 25 years ago, Anderson and colleague pointed to the high levels of depression and anxiety in RA patients.^18^ Therefore, it is clear that anxiety and depression provide an important indicator of the psychological distress experienced by patients with Rheumatoid Arthritis.

This study was done keeping in mind paucity of local data on prevalence of depression in patients with RA and to establish its association, primarily with disability and duration of disease. Studies have shown that health outcomes can be worse for RA patients with comorbid depression compared with those without depression.^19^ Hence earlier detection and treatment of depression can improve disease outcomes in this group of patients.

**Table-II:** Comparison between BDI score (severity of depression) * and M HAQ Score (disability).

		*M HAQ Score (disability)*				*Total*

		*0-0.3 normal*	*0.4 -1.2 mild*	*1.3-1.8 moderate*	*>1.8 severe*
BDI score(severity of depression)	0-9 no depression	36	29	2	0	67
		53.7%	43.3%	3.0%	0.0%	100.0%
		75.0%	43.9%	16.7%	0.0%	52.3%
	10-19 mild depression	12	32	5	1	50
		24.0%	64.0%	10.0%	2.0%	100.0%
		25.0%	48.5%	41.7%	50.0%	39.1%
	20 – 29 moderate depression	0	3	4	1	8
		0.0%	37.5%	50.0%	12.5%	100.0%
		0.0%	4.5%	33.3%	50.0%	6.3%
	>30 severe depression	0	2	1	0	3
		0.0%	66.7%	33.3%	0.0%	100.0%
		0.0%	3.0%	8.3%	0.0%	2.3%
Total		48	66	12	2	128
		37.5%	51.6%	9.4%	1.6%	100.0%
		100.0%	100.0%	100.0%	100.0%	100.0%

Patients with RA are two to four times more likely to suffer from depression compared to normal population.^20^ According to a local study, 65% of patients with RA have depression.^21^ Another study from this region reported depression in nearly 71.5% of patients with Rheumatoid Arthritis and there was a strong association between disease activity and the level of depression.^22^ According to our study, 47.7% of had depression of varying severity. This figure is in accordance with globally reported prevalence of depression in RA patients.

Recent data suggest that depression in RA patients is both common and under-recognized in the rheumatology setting, and may persist for years after diagnosis.^23^ As RA disease improves due to therapeutic interventions; depression and anxiety symptoms may potentially improve.^13^ Also patients with non-concordance with therapy were more frequently diagnosed with major depressive episode and tend to have higher BDI scores. They had significantly more disease activity according to patient-pain score and swollen joint counts.^24^ Though depression is prevalent and under recognized, at times it tends to be overestimated in patients with pain and disability.^25^

**Table:** Correlations

		*BDI score*	*Duration of Disease*	*Duration of Treatment*	*Number of Joint Damage*	*M HAQ Score (disability)*	*ESR*
BDI score	Pearson Correlation	1	.150	.080	.193*	.571**	-.122
	Sig. (2-tailed)		.092	.369	.029	.000	.169
	N	128	128	128	128	128	128
Duration of Disease	Pearson Correlation	.150	1	.862**	.754**	.299**	-.010
	Sig. (2-tailed)	.092		.000	.000	.001	.908
	N	128	128	128	128	128	128
Duration of Treatment	Pearson Correlation	.080	.862**	1	.600**	.214*	.010
	Sig. (2-tailed)	.369	.000		.000	.015	.906
	N	128	128	128	128	128	128
Number of Joint Damage	Pearson Correlation	.193*	.754**	.600**	1	.265**	.034
	Sig. (2-tailed)	.029	.000	.000		.002	.706
	N	128	128	128	128	128	128
M HAQ Score (disability)	Pearson Correlation	.571**	.299**	.214*	.265**	1	-.088
	Sig. (2-tailed)	.000	.001	.015	.002		.323
	N	128	128	128	128	128	128
ESR	Pearson Correlation	-.122	-.010	.010	.034	-.088	1
	Sig. (2-tailed)	.169	.908	.906	.706	.323	
	N	128	128	128	128	128	128

* Correlation is significant at the 0.05 level (2-tailed).

Physical disability and limited function, as measured by the Health Assessment Questionnaire, is a strong predictor of depression in patients with Rheumatoid Arthritis.^26^ In present study HAQ was found to be positively correlated with severity of depression according to BDI II scale. This underscores the importance of early recognition of depression as well as earlier detection of Rheumatoid arthritis. A multicenter prospective study comprising 641 patients with early RA suggested that psychological distress in very early RA is frequent and the HAQ-DI score is a predictor of depression and anxiety in these patients. A psychological evaluation in patients with early RA is important for further individual psychiatric diagnosis and management.^27^ Depression and anxiety in RA have been shown to be associated with increased pain and fatigue, reduced quality of life, and increased service use, disease activity and disability.^28^

According to Rathbun et al.^29^ onset of depression in RA patients is related to measures reported by the patient: pain, functional status, and global disease activity; and measures reported by providers, rather than biological markers. It implied that patients’ experience of their disease activity may be a precipitating factor of depression onset.

Our study also explored response of patients with RA and clinical depression when offered professional mental health care. About 82% of our patients with depression refused to see a psychiatrist. Almost 52.7% reported time constraint as a main limitation, 24.5% refused to acknowledge presence of depression and 22.8% gave no reason. It is also imperative to understand the reasons behind refusal to seek timely mental health care, particularly in our community where depression is an even bigger taboo than physical disability. Many studies have highlighted that Asians, amongst other ethnic groups are less likely to seek mental health care. The influence of Asian family and community stigma on mental health utilization and the lack of culturally appropriate mental health interventions is the main factor.^30^

The study had some worth mentioning pit falls the most important being gender bias in cohort selection as 95.3% of our patients were females. Also overlap of somatic symptoms with RA leads to overestimation of depression in such patients.

Our study emphasizes that apart from achieving early remission in RA the significance of timely diagnosis of co morbid depression and its treatment is equally important in the overall management of RA. It is prudent that clinicians screen patients for under lying depression and offer treatment to improve disease outcomes and achieve remission.

## CONCLUSION

Our study reinforces the fact that co-morbid conditions, like depression have significant impact on morbidity in RA and remain largely under recognized and under treated. With rapidly evolving management of RA and tighter control strategies being advocated to achieve remission early in the course of disease, it is pertinent that all patients be screened and treated for co-morbid conditions including depression to improve disease outcomes. Importance of early detection cannot be overemphasized however further research is required to understand the reluctance to seek mental health care in patients with chronic diseases.

### Authors Contribution

**AM** conceived, designed, collected data and wrote the manuscript.

**BS** conceived, designed, reviewed and edited the manuscript.

**AN** reviewed the manuscript.

**ZK** did data collection.

**AA** did statistical analysis.

## References

[1] Pytel A, Wrzosek Z Estimation of patient knowledge on Rheumatoid Arthritis in the range of their own disease –Preliminary study. Adv Clin Exp Med.2012;.

[2] Cross M, Smith E, Hoy D, Carmona L, Wolfe F, Vos T (2014). The global burden of rheumatoid arthritis:estimates from the Global Burden of Disease 2010 study. Ann Rheum Dis.

[3] Alam SM, Kidwai AA, Jafri SR Jour (2011). Epidemiology of rheumatoid arthritis in a tertiary care unit. Karachi. Pak. J Pak Med Assoc.

[4] Malaviya AN, Kapoor SK, Singh RR, Kumar A, Pande I (1993). Prevalence of rheumatoid arthritis in the adult Indian population. Rheumatol Int.

[5] (2006). W.H.O. Mental Health.

[6] Andrade L, Caraveo A (2006). Epidemiology of major depressive episodes:Results from the International Consortium of Psychiatric Epidemiology (ICPE) Surveys. Int J Methods Psychiatr Res.

[7] Kessler RC, Berglund P, Demler O (2003). The epidemiology of major depressive disorder:Results from the National Comorbidity Survey Replication (NCS-R). JAMA.

[8] Matcham F, Rayner L, Steer S (2013). The prevalence of depression in rheumatoid arthritis:a systematic review and meta-analysis. Rheumatology.

[9] Coty MB, Wallston KA (2008). Roles and well-being among healthy women and women with rheumatoid arthritis. J Adv Nurs.

[10] Matcham F, Ali S, Irving K, Hotopf M, Chalder T (2016). Are depression and anxiety associated with disease activity in rheumatoid arthritis? A prospective study. BMC Musculoskelet Disord.

[11] Matcham F, Rayner L, Hutton J, Monk A, Steel C, Hotopf M (2014). Self-help interventions for symptoms of depression, anxiety and psychological distress in patients with physical illness:a systematic review and meta-analysis. Clin Psychol Rev.

[12] Matcham F, Norton S, Scott DL, Steer S, Hotopf M (2016). Symptoms of depression and anxiety predict treatment response and long-term physical health outcomes in rheumatoid arthritis:secondary analysis of a randomized controlled trial. Rheumatol (Oxford).

[13] Kekow J, Moots R, Khandker R, Melin J, Freundlich B, Singh A (2011). Improvements in patient-reported outcomes, symptoms of depression and anxiety, and their association with clinical remission among patients with moderate-to-severe active early rheumatoid arthritis. Rheumatology (Oxford).

[14] Aletaha D, Neogi T, Silman AJ, Funovits J, Felson DT, Bingham CO (2010). 2010 Rheumatoid Arthritis Classification Criteria:An American College of Rheumatology/ European League Against Rheumatism Collaborative Initiative. Arthritis Rheum.

[15] Uhlig T, Haavardsholm EA, Kvien TK (2006). Comparison of the Health Assessment Questionnaire (HAQ) and the modified HAQ (MHAQ) in patients with rheumatoid arthritis. Rheumatology.

[16] Warmenhoven F, Rijswijk EV, Engels Y, Kan C, Prins J, Weel C (2012). The Beck Depression Inventory (BDI-II) and a single screening question as screening tools for depressive disorder in Dutch advanced cancer patients. Support Care Cancer.

[17] Pollard L, Choy EH, Scott DL (2005). The consequences of rheumatoid arthritis:quality of life measures in the individual patient. Clin Exp Rheumatol.

[18] Anderson KO, Bradley LA, Young LD, McDaniel LK, Wise CM (1985). Rheumatoid arthritis-review of psychological-factors related to etiology, effects, and treatment. Psychol Bull.

[19] Sheehy C, Murphy E, Barry M (2006). Depression in rheumatoid arthritis–underscoring the problem. Rheumatology.

[20] Margaretten M, Julian L, Katz P, Yelin E (2011). Depression in patients with rheumatoid arthritis:description, causes and mechanisms. J Clin Rheumtol.

[21] Amin A, Gadit M, Mugford G (2007). Prevalence of Depression among Households in Three Capital Cities of Pakistan:Need to Revise the Mental Health Policy. PLoS ONE.

[22] Imran MY, Khan SEA, Ahmad NM, Raja SF, Saeed MA, Haider II (2015). Depression in Rheumatoid Arthritis and its relation to disease activity. Pak J Med Sci.

[23] Hider SL, Tanveer W, Brownfield A, Mattey DL, Packham JC (2009). Depression in RA patients treated with anti-TNF is common and under-recognized in the rheumatology clinic. Rheumatology.

[24] Cabrera-Marroquín R, Contreras-Yáñez I, Alcocer-Castillejos N, Pascual-Ramos V (2014). Major depressive episodes are associated with poor concordance with therapy in rheumatoid arthritis patients:the impact on disease outcomes. Clin Exp Rheumatol.

[25] Poole H, White S, Blake C, Murphy P, Bramwell R (2009). Depression in chronic pain patients:prevalence and measurement. Pain Pract.

[26] Katz PP, Yelin EH (2001). Activity loss and the onset of depressive symptoms:do some activities matter more than others?. Arthritis Rheum.

[27] Bacconnier L, Rincheval N, Flipo RM, Goupille P, Daures JP, Boulenger JP (2015). Psychological distress over time in early rheumatoid arthritis:results from a longitudinal study in an early arthritis cohort. Rheumatology (Oxford).

[28] Schieir O, Thombs BD, Hudson M, Taillefer S, Steele R, Berkson L (2009). Symptoms of Depression Predict the Trajectory of Pain Among Patients with Early Inflammatory Arthritis:A Path Analysis Approach to Assessing Change. J Rheumatol.

[29] Rathbun AM, Harrold LR, Reed GW (2015). Temporal associations between the different domains of rheumatoid arthritis disease activity and the onset of patient-reported depressive symptoms. Clin Rheumatol.

[30] Augsberger A, Yeung A, Dougher M, Hahm HC (2015). Factors influencing the underutilization of mental health services among Asian American women with a history of depression and suicide. BMC Health Serv Res.

